# Daily Feeding of Fructooligosaccharide or Glucomannan Delays Onset of Senescence in SAMP8 Mice

**DOI:** 10.1155/2014/303184

**Published:** 2014-06-02

**Authors:** Sadako Nakamura, Naoyuki Kondo, Yoshitake Yamaguchi, Michiru Hashiguchi, Kenichi Tanabe, Chihiro Ushiroda, Miho Kawahashi-Tokuhisa, Katsuyuki Yui, Mana Miyakoda, Tsuneyuki Oku

**Affiliations:** ^1^Graduate School of Human Health Science, University of Nagasaki Siebold, 1-1-1 Manabino, Nagayo, Nagasaki 8512195, Japan; ^2^Institute of Food, Nutrition and Health, Jumonji University, 2-1-28 Sugasawa, Niiza, Saitama 3528510, Japan; ^3^Nagasaki City Hospital Organization, Nagasaki Municipal Hospital, 6-39 Shinchimachi, Nagasaki 8508555, Japan; ^4^Kyoto Koka Women's University, 38 Kadono-cho, Nishikyogoku, Ukyo-ku, Kyoto 6150882, Japan; ^5^Division of Immunology, Department of Molecular Microbiology and Immunology, Graduate School of Biomedical Sciences, Nagasaki University, 1-12-4 Sakamoto, Nagasaki 8528523, Japan

## Abstract

We hypothesized that daily intake of nondigestible saccharides delays senescence onset through the improvement of intestinal microflora. Here, we raised senescence accelerated mice prone 8 (SAMP8) on the AIN93 diet (CONT), with sucrose being substituted for 5% of fructooligosaccharide (FOS) or 5% of glucomannan (GM), 15 mice per group. Ten SAMR1 were raised as reference of normal aging with control diet. Grading of senescence was conducted using the method developed by Hosokawa, and body weight, dietary intake, and drinking water intake were measured on alternate days. Following 38 weeks of these diets we evaluated learning and memory abilities using a passive avoidance apparatus and investigated effects on the intestinal microflora, measured oxidative stress markers, and inflammatory cytokines. Continuous intake of FOS and GM significantly enhanced learning and memory ability and decelerated senescence development when compared with the CONT group. *Bifidobacterium* levels were significantly increased in FOS and GM-fed mice. Urinary 8OHdG, 15-isoprostane, serum TNF-**α**, and IL-6 were also lower in FOS-fed mice, while IL-10 in FOS and GM groups was higher than in CONT group. These findings suggest that daily intake of nondigestible saccharides delays the onset of senescence via improvement of intestinal microflora.

## 1. Introduction

Strains of senescence accelerated model mice (SAM) display features that render them suitable models of human aging. In particular, the SAM prone 8 (SAMP8) mouse is an appropriate model of human neurological aging [1, 2]. SAMP8 possess defects in learning and memory, emotional disorders, and a severe age-related impairment when assessed by the passive avoidance test [3, 4]. As these phenotypes are caused by various factors, including brain aging, neuroinflammation, and immunosenescence, the mechanisms that accelerate senescence in SAMP8 resemble those of human senescence [1, 2].

Intestinal microflora changes according to the aging, and the reduction of beneficial microbes and the increment of harmful microbes deteriorate the intestinal environment [5]. And intestinal microflora relates to colonic senescence via polyamine production and other factors [6]. SAMP8 cause quickly the change of intestinal microflora by accelerating senescence. Prebiotics such as nondigestible oligosaccharide which escape enzymatic digestion in the small intestine and are fermented by intestinal microbes, improve intestinal microflora, and contribute to human well-being [7–10]. Some prebiotics have been found to exert antioxidative and anti-inflammatory effects via improvement of intestinal microflora [11, 12]. Therefore, prebiotics may improve effectively the intestinal microflora of SAMP8 and delay the defects in learning and memory and emotional disorders. Antioxidative and anti-inflammatory agents present in food exacerbate the memory disorder and learning impairment in SAMP8 [13–15], decrease amyloid-*β* deposition [16], and mitochondrial dysfunction [17]. Ueda et al. [18] reported that the assessment by passive avoidance test in SAMP8 fed diet containing fish oil was better than that in SAMP8 fed high saturated fatty acids, because fish oil contains high polyunsaturated fatty acids. And it was reported that the lifespan of* C. elegans* was elongated by the inhibition of lipid peroxidation due to appropriate fish oil [19] and n-3 fatty acids decrease oxidative stress in ischemic rat hippocampus [20].

Fructooligosaccharide (FOS) is a safe, nondigestible oligosaccharide whose presence in the diet improves lipid and blood glucose metabolism and has an antioxidant and anti-inflammatory potentials [7, 10–12, 21]. Furthermore, the ingestion of nondigestible saccharides increases numbers of* Lactobacillus* and* Bifidobacterium* in the gut, and these microbes in turn decrease secretion of TNF-*α*, IL-1*β*, IL-6, and IL-17 [22]. Additionally, glucomannan (GM) is a soluble dietary fiber and nondigestible polysaccharide. Ingestion of GM stabilizes blood glucose concentrations and soluble dietary fiber is associated with decreased levels of oxidative stress [23]. Nondigestible saccharides, which escape the digestion by small intestinal enzymes, reach the large intestine and are fermented by intestinal microbes. And they are metabolized to short-chain fatty acids, gases, such as carbon dioxide, hydrogen, and methane, and the other metabolites.

Although many useful physiological functions of prebiotics have been previously described, interactions among various ingested nondigestible oligo/polysaccharides, their influence on delayed onset of aging-related diseases, and changes to the population of intestinal microflora are relatively unexplored. We hypothesized that daily ingestion of oligo/polysaccharide improves intestinal microflora (their metabolites also change), affects the agents of antioxidation and anti-inflammation, and leads to delay of the acceleration of senescence. Here, we examine the effect of daily feeding of nondigestible saccharides to SAMP8 on the onset of the typical phenotype reminiscent of age-related disease. Two nondigestible saccharides, the oligosaccharide FOS and the polysaccharide GM, were examined. The learning and memory abilities of SAMP8 were assessed by passive avoidance testing and were discussed from the viewpoint of the interaction among oxidative stress and inflammatory cytokines.

## 2. Materials and Methods

### 2.1. Animals, Diets, and Diet Feeding

A total of 45 male SAMP8 aged 4 weeks were purchased from SLC, Inc. (Shizuoka, Japan). The phenotypes reminiscent of onset of age-related disease in SAMP8 are learning and memory defect and emotional disorders [1, 2]. Ten male SAMR1 mice aged 4 weeks were used as a reference for normal onset of senescence. Mice were housed in individual plastic cages and kept at room temperature (23 ± 1°C); humidity was maintained at 50 ± 5% with a 12 h light/dark cycle (light, 8:00–20:00). Diets were slightly feed-restricted to facilitate equal food intake. Food and drinking water intake and body weight were measured on alternate days. Health status and fecal condition were observed daily. Mice were housed in a metabolic cage for 5 days before resection to collect and measure urine and fecal samples. Experiments were performed under the guidelines on the care and use of laboratory animals of University of Nagasaki Siebold.

Mice were fed with control diet for one week, and thereafter SAMP8 were assigned into three groups (15 mice per group) and fed as below for 38 consecutive weeks. The control diet group (CONT group; *n* = 15) was fed AIN93 diet [24]. The experimental groups were fructooligosaccharide group (FOS group; *n* = 15; Meiji Seika Kaisha Co., Ltd., Tokyo, Japan) and glucomannan group (GM group; *n* = 15; Shimizu Chemical Corp., Hiroshima, Japan). FOS was fed AIN93 diet in which the 5% sucrose was replaced with FOS and GM was fed AIN93 diet in which the 5% sucrose was replaced with GM. Ten SAMR1 (R1 group), which were raised as the reference group to show the normal aging, were fed control diet. Cellulose in AIN93 was replaced with *β*-corn starch to exclude the effect of cellulose on intestinal microflora.

FOS is a typical nondigestible oligosaccharide whose chemical structure is a mixture of 1^F^-(*β*-fructofuranosyl)_*n*−1_-sucrose, in which n varies from 2 to 4 (e.g., 2, 1-kestose (GF2), 28%; 3, nystose (GF3), 60%; 4, 1^F^-*β*-fructofuranosyl nystose (GF4), 12%). GM is a nondigestible polysaccharide, with an average molecular weight of 200,000. GM is used as a food ingredient and additive.

### 2.2. Assessment of Senescence Acceleration in SAMP8 

#### 2.2.1. Evaluation of Learning and Memory Disorder Using Passive Avoidance Test

A step-through passive avoidance apparatus (passive avoidance chamber LE872, Bio Research Center, Inc., Aichi, Japan) with light (25 × 25 × 30 cm) and dark (7 × 7 × 15 cm) compartments and the ShutAvoid system (Bio Research Center, Inc., Aichi, Japan) were used to evaluate learning and memory ability. The light compartment was illuminated with 300 lux and connected to a next dark compartment with an automatic electric door. The passive avoidance response was evaluated by the difference in retention and acquisition time. Since the onset of learning and memory disorder is typically observed at 4 months of age [1, 2, 25], assessment was performed at 13 weeks of feeding (before onset) for 5 out of 10 SAMR1 mice and for 6 out of 15 SAMP8 in each group. And the assessment was operated at 37 weeks of feeding for 5 SAMR1 and for 9 out of 10 SAMP8 in each group. These mice had not been used in the assessment trial at 13-week feeding.

An evaluation trial of learning and memory was conducted as follows [25]. (1) Adaptation trial: a mouse was placed in the light compartment facing away from the closed division door. The door was opened after 180 sec allowing the mouse free movement for 420 sec. (2) Acquisition trial: a mouse was placed in the light compartment facing away from the closed division 24 h after the adaptation trial. The door was opened from 60 to 180 sec after the mouse was placed in the light compartment. When the mouse stepped into the dark compartment, the division door was closed and the mouse was exposed to a punishing electrical shock (0.5 mA, 3 sec). Latency time A was defined as the time from which the door had opened to the time when a mouse entered into the dark compartment. (3) Retention trial: the same experimental procedure as the acquisition trial was performed 24 h after the acquisition trial, with the time between door opening and mouse entry to the dark compartment being defined as latency time R. We evaluated the learning and memory ability using the latency time R. It was considered that the mice whose latency time R is longer could maintain the learning and memory of the electrical shock.

#### 2.2.2. Grading Score Using the Hosokawa Method

Grading score consisted of eight parameters modified from the Hosokawa method [26]. We assessed reactivity, passivity, glossiness, coarseness, hair loss, ulceration, corneal opacity, and lordokyphosis by a single blinded method at 2, 4, 5, 6, 7, 8, and 9 months of age, and all mice were operated repeatedly.

### 2.3. Analysis of Profiles of Cecal Bacterial and Bacterial Enzymes

The resection was done for mice which had been used for passive avoidance test at 37 weeks of feeding, so the final numbers of mice for the analysis of organs and tissues weight, profiles of cecal bacteria and bacterial enzymes, urine, brain homogenate, and sera were as follows: R1 group: *n* = 5; CONT group: *n* = 7; FOS group: *n* = 8; GM group: *n* = 9. Two out of 9 mice in CONT group and one out of 9 mice in FOS group died at the 38 weeks of feeding.

To detect differences in populations of intestinal microbes, mice cecal contents were cultured based on Mitsuoka's method [5]. The cecum was removed keeping under anaerobic condition and transferred into anaerobic chamber. The cecal contents were weighed and homogenized with buffer solution prepared for anaerobic incubation. Media for culture were selective for genus of* Bifidobacterium*,* Lactobacillus*,* Bacteroides*, and* Clostridium*, and total anaerobic microbes were cultured using BL medium. Media were cultured under anaerobic condition at 37°C for 48 h and* Bifidobacterium* was cultured under the same condition for 72 h. The bacterial counts were calculated colony forming units per 1 g of dried cecal matter.

After feces were homogenized with 9 volumes of phosphate buffered saline (PBS), the homogenate was ultracentrifuged at 105,000 ×g for 30 min, 4°C, and the supernatant collected was stored at −20°C. The assay of *β*-glucuronidase and *β*-glucosidase activity in fecal supernatant was carried out according to the method of Freeman [27] and Gråsten et al. [28]. Substrates of *β*-glucuronidase and *β*-glucosidase activity were measured using 4 mM of* p*-nitrophenyl-*β*-D-glucuronide and 4 mM of* p*-nitrophenyl-*β*-D-glucopyranoside, respectively. Mixed medium of sample (0.5 mL) and substrate (0.5 mL) was incubated in duplicate at 37°C for 30 min. The reaction was stopped with 0.5 mL of 1 M sodium carbonate solution to add in incubation mixture and the absorbance was read at 415 nm by spectral photometer (UVmini-1240, Shimadzu Co., Ltd., Kyoto, Japan). The specific activity of enzyme was calculated as *μ*moles of hydrolyzed substrate per mg of protein per 1 hour.

### 2.4. Determination of Oxidative Stress and Antioxidant Markers in Urine, Brain, and Sera

Urine collected was centrifuged at 12,000 ×g, for 15 min at 4°C to be free from microbes. Urinary 8-hydroxy-2′-deoxyguanosine (8OHdG) and 15-isoprostane were measured by 8OHdG ELISA kit and urinary 15-isoprostane F_2t_ ELISA kit, respectively (Nikken Seil Co., Ltd., Shizuoka, Japan). Mouse brain was removed as the Mathis method [29] and the homogenate was prepared with PBS. After centrifugation, the supernatants were stored at −20°C. Malondialdehyde (MDA) in brain supernatant was measured using an MDA assay kit by the TBARS method (Nikken Seil Co., Ltd.). Sera were obtained by centrifugation at 15,000 ×g, for 5 min at 4°C. Oxidative stress and antioxidant potential were measured by a free radical detector and using kit (Free, Free Radical Elective Evaluator, Wismar Co., Ltd., Tokyo, Japan). Oxidative stress was evaluated by hydroperoxide using reactive oxygen metabolites test kit (d-ROM, Wismar), and antioxidant potential was evaluated by reducing activity from Fe^3+^ to Fe^2+^ using biological antioxidant potential test kit (BAP, Wismar).

### 2.5. Analysis of Serum Cytokines

Interleukin- (IL-) 2, IL-6, IL-10, and IL-17, interferon (IFN)-*γ*, and tumor necrosis factor- (TNF-) *α* in serum were measured by a cytometric bead array method (CBA mouseTh1/Th2/Th17 Cytokine kit, Becton Dickinson Biosciences, USA) using a FACSCantoII (BD Biosciences, USA) and analyzed using FCAP software (BD Biosciences, USA).

### 2.6. Protein Determination

Protein concentration in brain homogenates and cecal supernatant were determined by the Bradford method [30] using bovine serum albumin as a standard.

### 2.7. Calculation and Statistical Analysis

Data were calculated as mean and standard deviation (SD), differences were compared using ANOVA and Tukey's* post hoc* tests following the normal distribution test using SPSS ver. 21, and a *P* value less than 0.05 was considered significant.

## 3. Results

### 3.1. Growth, Food Intake, and Diet Efficiency

Table 1 shows the total food intake for 38 weeks, initial and final body weight, body weight gain, and diet efficiency in all raised mice. The numbers of mice in each group were as follows: R1 group: *n* = 10, CONT group: *n* = 13, FOS group: *n* = 14, and GM group: *n* = 15, respectively.

No significant difference in final body weight was observed among the four groups. Total food intake in CONT, FOS, and GM groups was not significantly different but much more significant than that in R1 group as a reference group (*P* < 0.05). Final body weight in GM was the lightest of the four groups and the dietary efficiency of the GM group was significantly lower than that of the other 3 groups (*P* < 0.05).

### 3.2. Weights of Organs and Tissues

Table 2 displays the organs and tissues weight per 100 g of body weight in mice following 38 weeks after feeding of each diet, R1 group (*n* = 5), CONT group (*n* = 7), FOS group (*n* = 8), and GM group (*n* = 9). Significant differences were observed in heart and lungs (*P* < 0.05), but they were within normal ranges. The weights of colon in FOS and GM groups were significantly heavier (*P* < 0.05) than that in R1 group and tended to be heavier than that in CONT group. The epididymal adipose tissues in SAMP8 groups were significantly lighter than that in R1, respectively (*P* < 0.05).

### 3.3. Effect of Feeding FOS or GM on the Grading Score

**Figure 1:**
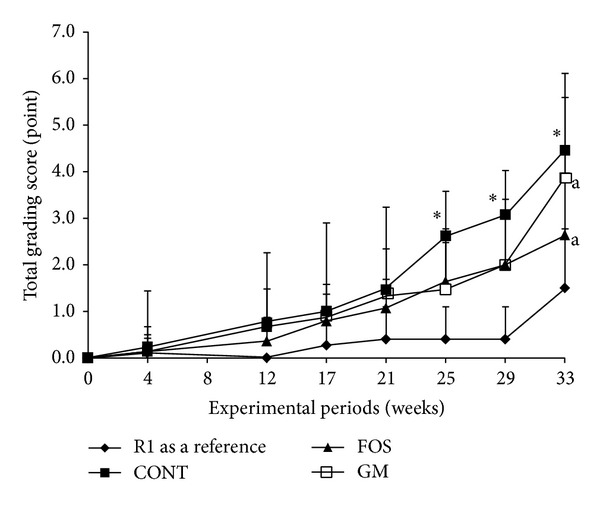
Effects of FOS or GM feeding on grading score of SAMR8 during feeding period. Values were expressed as mean ± SD. R1, SAMR, *n* = 10; CONT, control diet, *n* = 15; FOS, 5% of fructooligosaccharide diet, *n* = 15; GM, 5% of glucomannan diet, *n* = 15. ^∗^Significant differences were evaluated versus CONT by one-way ANOVA and Tukey's* post hoc* test, at *P* < 0.05. a: significant difference between FOS and GM by one-way ANOVA and Tukey's* post hoc* test, at *P* < 0.05.

Profiles of the Hosokawa method grading score during 33 weeks of feeding are shown in Figure 1. The grading score in R1 group (*n* = 10) was very low, because the senescence in R1 group is normal. The grading score in CONT group (*n* = 15) was significantly higher than that in FOS (*n* = 15) and GM groups (*n* = 15) from 25 weeks after feeding (*P* < 0.05). And after 33 weeks of feeding, grading score in FOS group was significantly lower than that in CONT group (*P* < 0.05), but that in GM group was not significantly different from CONT group.

### 3.4. Evaluation of Learning and Memory Ability

**Figure 2:**
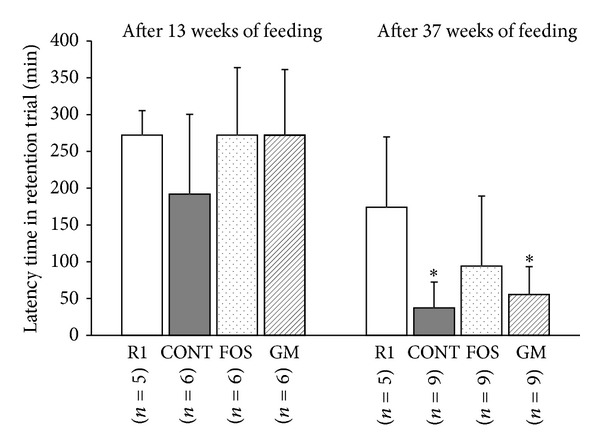
Effects of FOS or GM feeding on learning and memory performance in SAMP8 after 13 weeks and 37 weeks of feeding. R1, SAMR1, and control diet; CONT, control diet; FOS, 5% of fructooligosaccharide diet; GM, 5% of glucomannan diet. ^∗^Significant differences versus SAMR1, respectively, at *P* < 0.05 by ANOVA and Tukey's* post hoc* test.

The latency time R is shown in Figure 2. After 13 weeks of feeding, no significant difference was observed among the four groups (*n* = 5 in R1, *n* = 6 in CONT, FOS, and GM). However, after 37 weeks of feeding, the latency times R in CONT (*n* = 9) and GM (*n* = 9) groups were significantly shorter than that in R1 group (*n* = 5) (*P* < 0.05). But the latency times R in FOS group (*n* = 9) were not significantly different from that in R1 group. The deviation of latency time in FOS group was large because the mice which did not enter the dark compartment were involved in FOS group.

### 3.5. Effect on the Population of Cecal Microbes, Weight of Cecal Tissue and Content, and *β*-Glucosidase and *β*-Glucuronidase Activities

Table 3 shows the anaerobic bacterial counts per 1 g of cecal dry matter in selective medium. Total bacterial counts in FOS (*n* = 8) and GM (*n* = 9) groups were much more than that in CONT (*n* = 7) group, but it was not significant.* Bifidobacterium* genus in FOS group was significantly increased than that in CONT and R1 groups (*P* < 0.05).

**Figure 3:**
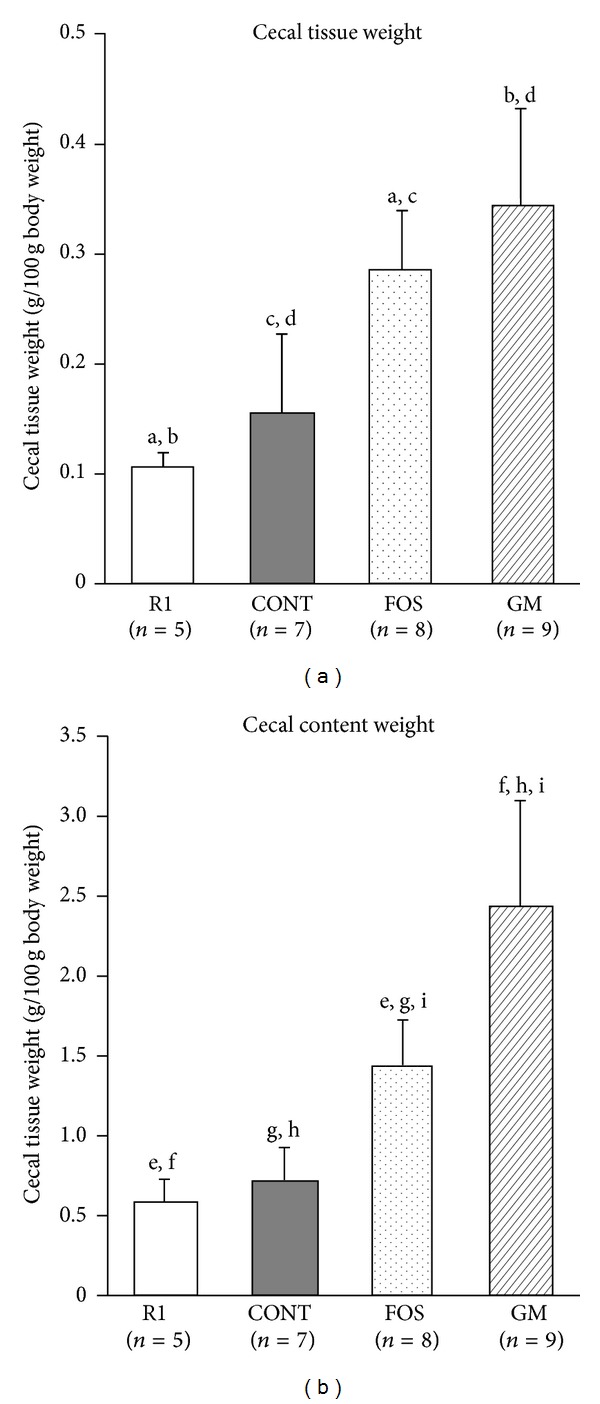
Weights of cecal tissue and content in SAMP8 fed diet containing FOS or GM at 38 weeks after feeding. Values were expressed as mean ± SD. R1, SAMR1, and control diet; CONT, control diet; FOS, 5% of fructooligosaccharide diet; GM, 5% of glucomannan diet. a–i: significant differences were evaluated by ANOVA and same superscripts were significantly different by Tukey's* post hoc* test, at *P* < 0.05.

The weights of cecal tissue and content in FOS and GM groups were significantly higher than those in CONT and R1 (*n* = 5) groups (*P* < 0.05; Figure 3).

**Figure 4:**
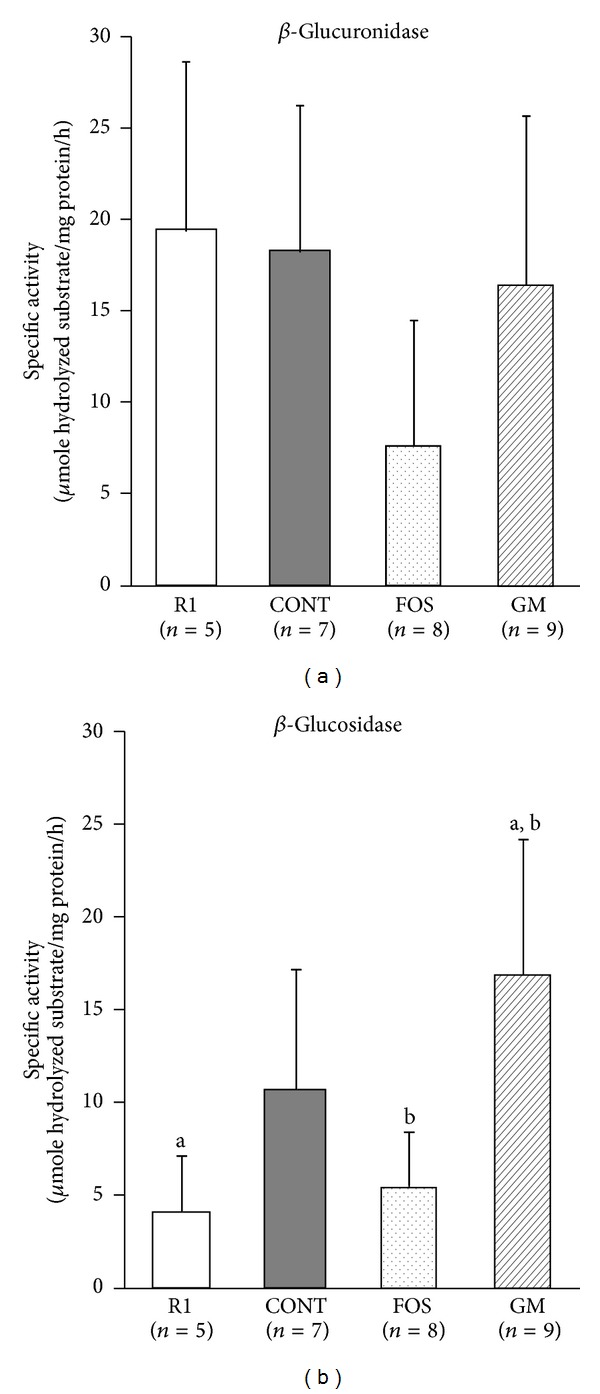
Effects of FOS or GM feeding on microbial enzyme activities in feces at 38 weeks after feeding. Values were expressed as mean ± SD. R1, SAMR1, and control diet; CONT, control diet; FOS, 5% of fructooligosaccharide; GM, 5% of glucomannan. a, b: significant differences were evaluated by one-way ANOVA and same superscripts were significantly different by Tukey's* post hoc* test, at *P* < 0.05.

The activity of *β*-glucuronidase tended to be lower in FOS group and *β*-glucosidase activity was significantly higher in GM group than in R1 and FOS groups (*P* < 0.05; Figure 4).

### 3.6. Differences in Oxidative Stress and Antioxidant Markers

**Figure 5:**
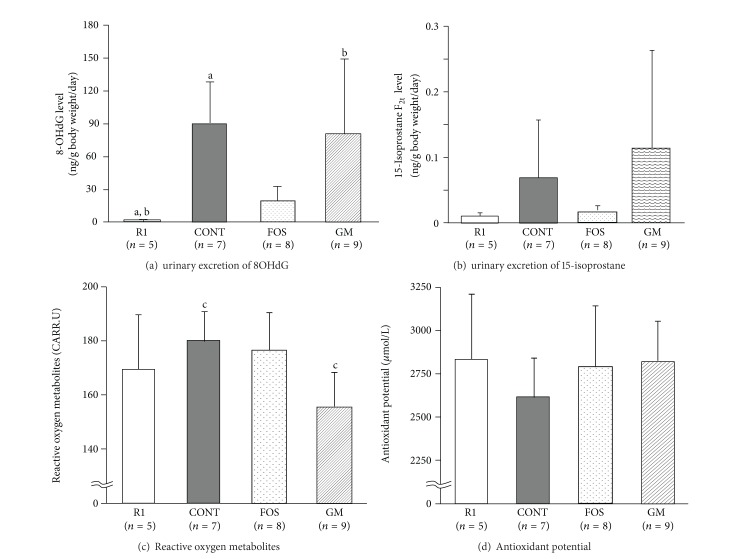
Effect of FOS or GM feeding on oxidative stress and antioxidant markers in urine and serum at 38 weeks after feeding. Values were expressed as mean ± SD. R1, SAMR1, and control diet; CONT, control diet; FOS, 5% of fructooligosaccharide diet; 5% of GM, glucomannan diet. a–c: significant differences were evaluated by ANOVA and same superscripts were significantly different by Tukey's* post hoc* test, at *P* < 0.05.

**Figure 6:**
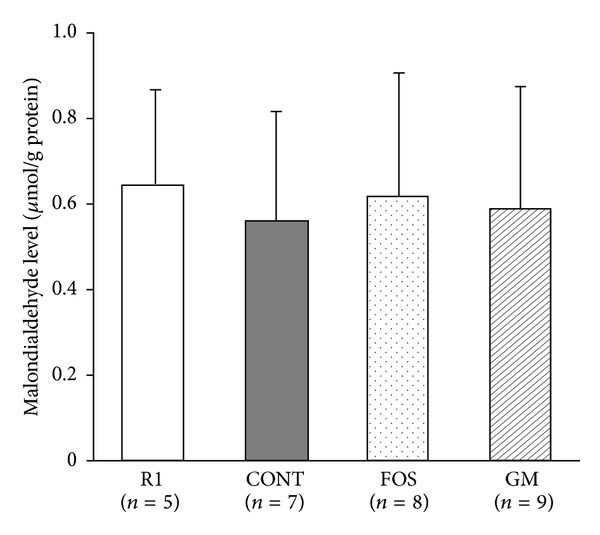
Effect of FOS or GM feeding on cerebral malondialdehyde at 38 weeks after feeding. Values were expressed as mean ± SD. R1, SAMR1, and control diet; CONT, control diet; FOS, 5% of fructooligosaccharide diet; GM, 5% of glucomannan diet. There was no significant difference among SAMR1 and SAMP8 groups by ANOVA.

Levels of oxidative stress markers in urine are shown in Figures 5(a) and 5(b), oxidative stress and antioxidant potential marker in serum are shown in Figures 5(c) and 5(d), and MDA levels in brain homogenate are shown in Figure 6. The numbers of mice were as follows: R1 group: *n* = 5, CONT group: *n* = 7, FOS group: *n* = 8, and GM group: *n* = 9, respectively.

Urinary excretion of 8OHdG (Figure 5(a)) in FOS group was not significantly different versus R1 group which shows normal aging, while that in CONT and GM groups was significantly higher than that in R1 group (*P* < 0.05). Urinary excretion of 15-isoprostane (Figure 5(b)) in CONT and GM groups tended to be higher, but this was not significant. Furthermore, oxidative stress marker (d-ROM, Figure 5(c)), which reflects total amount of hydroperoxide, was significantly lower in GM group than CONT group and antioxidant potential (BAP, Figure 5(d)) in CONT group tended to be lower among the four groups. MDA levels in brain homogenate were not significantly different among the four groups (Figure 6).

### 3.7. Profiles of Cytokines in Serum

**Figure 7:**
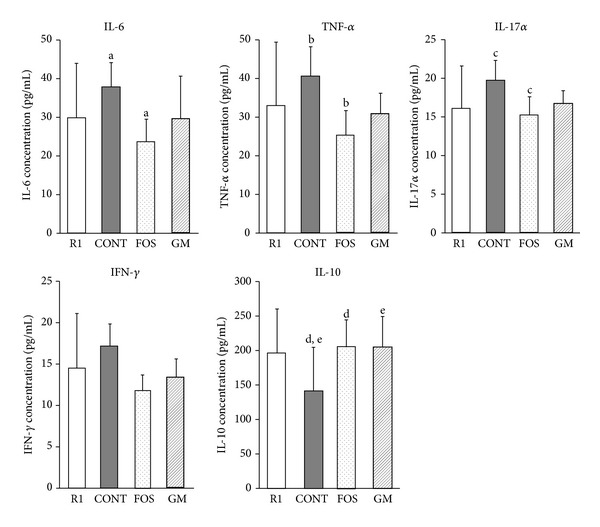
Difference in serum inflammatory cytokines at 38 weeks after feeding. Values were expressed as mean ± SD. R1, SAMR1, and control diet, *n* = 5; CONT, control diet, *n* = 7; FOS, 5% of fructooligosaccharide diet, *n* = 8; GM, 5% of glucomannan diet, *n* = 9. a–e: significant differences were evaluated by ANOVA and same superscripts were significantly different by Tukey's* post hoc* test, at *P* < 0.05.

Levels of IL-6, TNF-*α*, and IL-17 were significantly lower in FOS group than in CONT group (*P* < 0.05; Figure 7). IL-10 in both FOS and GM groups was significantly higher than in CONT group (*P* < 0.05; Figure 7).

## 4. Discussion

Here, we describe how the accelerated senescence and the onset of learning and memory disorders observed in SAMP8 can be delayed by daily feeding of 5% FOS or 5% GM in the diet. Cytokine profiles and oxidative stress markers are modified by metabolites produced by intestinal microbes acting upon nondigestible saccharides. Our further investigations suggest that this phenomenon is related to the modification of oxidative stress and cytokines via changes to the intestinal microflora.

FOS and GM are nondigestible saccharides that are not digested in the small intestine and reach the large intestine, where they are fermented by intestinal microbes [7–10]. In this study, the weights of cecal tissues, contents, and colon were heavier in FOS and GM groups. These changes were certainly observed by the feeding of nondigestible saccharide due to the hyperplasia of epithelial cells [31]. The bacterial counts in cecum increased in FOS and GM groups, and cecal microflora population was altered following feeding of FOS and GM. It has been reported that the activities of *β*-glucuronidase and *β*-glucosidase are decrease by the feeding of FOS due to the increasing of* Bifidobacterium* and* Lactobacillus* genus and the decreasing of* Clostridium* genus. In this study, the decreased activities of *β*-glucuronidase and *β*-glucosidase in FOS group compared with CONT group may result from changes in* Bifidobacterium* populations, although the bacterial counts of* Clostridium* genus were not significantly different. On the other hand, in the mice fed GM decreased activity of these enzymes was not observed. The reason was not dissolved in this study, because in this time we cultured* Clostridium* genus bacteria and did not separately detect the specific species of* Clostridium* which produced these enzymes [32].

Learning and memory ability are affected in SAM by intake of some food components. For example, Umezawa et al. [33] and Kohno et al. [34] have shown that energy restriction elongates lifespan in SAM, and unsaturated fatty acids, such as n-3 fatty acids in fish oil, also affect lifespan and learning and memory abilities [18]. Spirulina strains lessen the severity of learning and memory disorders and are reported to decrease amyloid-*β* deposition in the brain [16]. Further, as curcumin [17] and nobiletin [13], resveratrol [35] prevents oxidative stress-induced damage, activates AMPK, and increases lifespan in SAMP8. In this study, the levels of urinary 8OHdG and 15-isoprostane in FOS-fed mice were lower in comparison with those in CONT and GM groups and the same as those in R1 group. Although antioxidant potential in serum and MDA in brain homogenate were not significantly different across the four groups, antioxidant potential in CONT group tended to be lower among 4 groups. In our preliminary trial, we observed that the activity of glutathione reductase was higher in FOS group and glutathione disulfide in FOS and GM groups was not significantly different than that in R1 group, although that in CONT group tended to be higher. These results suggested that the oxidative stress related to the assessment of learning and memory ability in SAMP8. But we think that further studies in terms of the oxidative stress, antioxidant potential, and their reason are required.

On the other hand, hydrogen gas is produced when intestinal microbes ferment FOS and GM [36, 37] and it was absorbed from the gastrointestinal tract by diffusion. Hydrogen gas absorbed is carried to organs and tissues via blood circulation. A part of hydrogen produced was excreted with flatus, and the remaining gas was finally excreted into end-expiratory gas. We have already clarified that the excretion of hydrogen in end-expiratory gas was increased surely by the ingestion of nondigestible saccharide in a dosage manner [36, 37]. Recently, hydrogen gas which is exogenously administered to the patients acts to prevent the progression to severe oxidative stress [38–40]. Oxidative stress acts to stimulate early stage of amyloid-*β* protein deposition and *γ*-secretase activity [41]; deposited amyloid-*β* induces oxidative stress [42]. So, the reduction of oxidative stress relates to delay of the onset of learning and memory disorder [43]. Our next hypothesis is that the hydrogen gas was produced by the fermentation of nondigestible saccharide via intestinal microbes, transferred to brain via blood stream, and acts as antioxidant agent.

The inflammatory cytokines TNF-*α*, IL-6, and IL-1*β* upregulate amyloid precursor protein and *γ*-secretase, causing an accumulation of amyloid-*β* protein [41–43]. In this study, levels of IL-6, TNF-*α*, and IL-17 in FOS group were significantly lower than in CONT group and tended to be lower in GM group. The ingestion of nondigestible saccharides alters intestinal microflora, resulting in decreased production of inflammatory cytokines, and ingestion of nondigestible saccharide decreases the production of TNF-*α* and IL-1*β*. Alzheimer's disease develops with accumulation of amyloid-*β* protein, and concentrations of anti-inflammatory cytokines are related to the status of this disease [2, 41, 42]. Therefore, one factor involved in the delayed acceleration of learning and memory disorder in FOS and GM groups is the decreased serum concentration of inflammatory cytokines.

Although the results of the passive avoidance test in GM group were similar to those in FOS group, antioxidative stress markers and the profile of inflammatory cytokines were not so markedly improved in comparison with FOS group. FOS is low-molecular oligosaccharide and is easily fermented by intestinal microbes. However, GM is a large molecular weight nondigestible polysaccharide and exhibits less fermentability by intestinal microbes than FOS. Consequently, the degree of fermentation by intestinal microbes may affect the concentration of cytokines and antioxidative stress markers. Furthermore, the final body weight of GM group was the lightest of the four groups, and dietary efficiency was significantly lower in this group. Restriction of dietary intake prolongs lifespan in SAMP8 [33, 34] and antioxidant agents such as resveratrol act similarly [35]. As the available energy of dietary fibers is between 0 and 2 kcal per gram and that of FOS is 2 kcal per gram [44, 45], actual intakes of total energy in FOS and GM groups were lower than that in R1 and CONT groups, though this difference was not significant. It remains possible that the slightly lower energy intake affects the improvement of learning and memory abilities in GM group. Although the previously identified mechanism for this phenomenon has not been clarified in this study, we suspect that FOS and GM may act via different pathways to achieve a similar end.

## 5. Conclusions

The feeding of a diet containing FOS or GM in place of sucrose slowed the acceleration of senescence and delayed the onset of learning and memory disorders typically seen in SAMP8. The intestinal microflora in mice fed FOS or GM was different compared with control diet groups. Furthermore, oxidative stress markers and inflammatory cytokine levels were significantly lower in FOS and GM-fed mice. These results strongly suggest that daily feeding of nondigestible oligosaccharide and dietary fiber decelerate the onset of aging-related diseases via improvement of the intestinal microflora. This study therefore contributes valuable knowledge to the understanding of senescence.

## Figures and Tables

**Table 1:** Food intake, body weight gain, and diet efficiency of SAMR1 and SAMP8 fed diet containing FOS or GM.

	Total food intake (g)*	Initial body weight (g)	Final body weight (g)	Body weight gain (g)*	Diet efficiency (%)*
R1 (*n* = 10)	1018.2 ± 55.9^a^	21.8 ± 1.1	39.7 ± 7.9	18.0 ± 7.5	1.8 ± 0.4
CONT (*n* = 13)	1252.4 ± 84.1	20.8 ± 1.3	39.3 ± 9.9	18.5 ± 10.6	1.5 ± 0.9
FOS (*n* = 14)	1167.1 ± 50.5	20.5 ± 1.5	41.0 ± 6.4	20.3 ± 5.9	1.7 ± 0.5
GM (*n* = 15)	1243.1 ± 79.1	20.5 ± 1.5	36.2 ± 7.2	15.7 ± 7.7	1.3 ± 0.7^b^

Values were expressed as mean ± SD. R1, SAMR1, and control diet; CONT, control diet; FOS, 5% of fructooligosaccharide diet; GM, 5% of glucomannan diet. *Total food intake, and body weight gain, diet efficiency were calculated based on the feeding periods during 38 weeks. ^a^R1 was significantly different versus CONT, FOS, and GM, respectively, at *P *< 0.05 by Tukey's *post hoc* test. ^b^GM was significantly different than R1, FOS, and GM, respectively, at *P *< 0.05 by Tukey's *post hoc* test.

**Table 2:** Relative weight of whole brain, right hemisphere, left hemisphere, colon, organs, and adipose tissues in SAMP8 at 38 weeks after feeding.

	R1 (*n* = 5)	CONT (*n* = 7)	FOS (*n* = 8)	GM (*n* = 9)
Whole brain	1.22 ± 0.13	1.24 ± 0.23	1.24 ± 0.13	1.29 ± 0.12
Right hemisphere	0.24 ± 0.03	0.29 ± 0.10	0.29 ± 0.07	0.32 ± 0.06
Left hemisphere	0.24 ± 0.01	0.31 ± 0.10	0.31 ± 0.09	0.33 ± 0.07
Liver	5.92 ± 0.98	7.70 ± 2.19	5.61 ± 0.79	7.54 ± 3.20
Heart	0.41 ± 0.04^a^	0.45 ± 0.05	0.45 ± 0.03	0.50 ± 0.07^a^
Spleen	0.24 ± 0.06	0.26 ± 0.05	0.32 ± 0.18	0.33 ± 0.12
Lungs	0.47 ± 0.05^b,c^	0.57 ± 0.13	0.61 ± 0.09^b^	0.65 ± 0.08^c^
Colon	0.11 ± 0.01^d,e^	0.16 ± 0.07	0.28 ± 0.05^d^	0.35 ± 0.08^e^
Kidneys	1.47 ± 0.15	1.48 ± 0.47	1.30 ± 0.08	1.73 ± 0.31
Epididymal adipose tissue	4.06 ± 1.53^f,g,h^	1.44 ± 1.01^f^	2.43 ± 0.90^g^	1.28 ± 0.89^h^
Perirenal adipose tissue	1.77 ± 0.48	1.69 ± 1.05	1.88 ± 0.44	1.17 ± 0.98

Unit: g/100 g of body weight. Values were expressed as mean ± SD. R1, SAMR1, and control diet; CONT, control diet; FOS, fructooligosaccharide diet; GM, glucomannan diet. ^a–h^There were significant differences between same letters, at *P *< 0.05 by Tukey's *post hoc* test.

**Table 3:** Profiles of bacterial count in cecal at 38 weeks of feeding.

	R1 (*n* = 5)	CONT (*n* = 7)	FOS (*n* = 8)	GM (*n* = 9)
*Bifidobacterium* genus	3.0 ± 2.0	3.2 ± 1.6	14.6 ± 8.5^a^	12.5 ± 9.7
*Lactobacillus* genus	12.1 ± 10.6	3.3 ± 3.6	4.7 ± 3.7	6.6 ± 8.5
*Bacteroides* genus	3.2 ± 2.6	1.5 ± 2.5	5.4 ± 7.0	3.9 ± 3.7
*Clostridium* genus	11.9 ± 1.0	8.9 ± 6.7	32.8 ± 38.9	31.4 ± 28.7

Unit: ×10^8^ colony forming unit/1 g of cecal dry matter. Values were expressed as mean ± SD in selective medium. R1, SAMR1, and control diet; CONT, control diet; FOS, fructooligosaccharide diet; GM, glucomannan diet. ^a^Significantly different from R1, CONT, and GM, at *P *< 0.05 by Tukey's *post hoc* test.
